# Why district assemblies disburse resources to district health systems for service delivery at district level in the context of decentralization: a comparative study of two districts in the Volta Region of Ghana

**DOI:** 10.3389/fpubh.2023.1136210

**Published:** 2023-08-14

**Authors:** Andrews Ayim, Irene Akua Agyepong, Nana Enyimayew

**Affiliations:** ^1^Policy, Planning, Monitoring and Evaluation Division, Ghana Health Service, Accra, Ghana; ^2^Dodowa Health Research Center, Research and Development Division, Ghana Health Service, Dodowa, Ghana; ^3^Public Health Faculty Ministries, Ghana College of Physicians and Surgeons, Accra, Ghana

**Keywords:** decentralization, devolution, district assemblies, district health systems, financial disbursement

## Abstract

**Objectives:**

To explore why the District Assembly disburses financial and other resources to the District Health System.

**Design:**

Multiple case study with a single unit of analysis (holistic) using quantitative and qualitative methods of data collection involving a desk review, analysis of routine health management information system data and key informant interviews.

**Setting:**

Two districts in the Volta Region of Ghana.

**Participants:**

Twelve key officials of each district assembly and the district health system (24 total) who had worked in the district at least a year or more.

**Interventions:**

None.

**Results:**

Both District Assemblies had moderate decision space which was influenced by their capacity, power and contextual factors like politics, economics, legal and situational factors. Disbursement of financial and other resources to the District Health Systems was influenced by financial capacity, use of power by stakeholders, context and the decision space of the District Assembly. Political actors appeared to have more power in resource disbursement decision making than community members and technocrats in a context of resource constraints and inadequate funding. The funding available was used predominantly for capital investments, mainly construction of Community Based Health Planning and Services (CHPS) compounds.

**Conclusion:**

It is important to make policies that will regulate the relative power among the political appointees like the District Chief Executives (DCEs), public and civil servants in decentralized departments and agencies and Community members to make resource disbursement more sensitive to communities and decentralized departments.

## Introduction

Decentralization is a strategy for transferring authority and responsibility from central to sub-national levels of government. Sometimes, governments only adopt the concept in theory and fail to delegate power to the districts and regions (1). Bossert (2) made the argument that decentralization will improve the delivery of services only when an appropriate degree of discretion is combined with adequate institutional capacities to make choices consistent with good health sector performance and accountability of those choices to local health needs and priorities. Literature on accountability argues that actively involving local democratic structures and civil society in decision-making will make public services delivery more responsive to local health needs and reduce the risk of elite capture (3, 4). Work done in Indonesia in 2006 where there had been a radical political, administrative and fiscal decentralization with the delivery of health services becoming the responsibility of district governments revealed that district governments were reliant on the central government for as much as 90% of their revenue even though public funding for health services more than doubled between 2001 and 2006. Key financial decisions were still made by the central government. It was concluded that in contrast to the promise of decentralization there has been little increase in the potential for discretion at the district level in managing public funds for health (5). This findings was in contrast to the belief that allowing local communities and regional entities to manage their own affairs, and through facilitating closer contact between central and local authorities, effective systems of local governance enable responses to people’s needs and priorities to be heard, thereby ensuring that government interventions meet a variety of social needs (6). Bossert’s work in 2014 on Empirical studies on decentralization investigated how institutional capacity helps to make good decisions and accountability to local elected officers as well as the interaction between decision space, capacity and accountability. He described decentralization as a set of rules about local choice and incentives that the central authorities use to encourage local decision makers to make choices that are likely to achieve the objectives of the central authorities. The approach defined the ‘decision space’ or local discretion allowed by the central government for functions and sub-functions about financing, service delivery, human resources and governance. It emphasized that decentralization is fundamentally about shifting choice from central authorities to local authorities but the choice allowed is not a single block but rather a range of discretion allowed over different functions. This is a more realistic way of viewing the complexity of real experience than the usual dichotomous descriptions in which systems are defined as decentralized or centralized. In the comparative decision space for Ghana, Zambia, Uganda and Philippines, the study found that Ghana had a moderate range of choice in financing function and Philippines had a wide range of choice (7). In the various areas of finance function, Zambia had a narrow range of choice in the area of sources of Revenue but had moderate choices in the area of expenditure and income from fees. The study realized that the formal legal and regulatory rules about decision space did not really define the actual practice of officials. So, though decentralization offers many opportunities, (8) concludes that to work properly, a decentralized system needs well-defined rules and enforcement. Otherwise, decentralization becomes a risky venture, particularly in poor developing countries, such as most of the African countries, where democratic institutions are fragile, and capacity is weak (8, 9). Concluded in their work that decentralization has not fulfilled its promise. They explained that after over 16 years after the adoption of the constitution, municipal governance in South Africa is in a state of paralysis, service delivery failure, and dysfunction. Hardly a day goes by when the country does not experience a “service delivery” protest somewhere.

A World Bank study in 2012 titled “A Health Sector in Transition to Universal Coverage in Ghana” suggested that there should be a Decentralization Policy and Legal Framework in health, on either moving the agenda to support the devolution and/or to stay with the current modality of decentralization through delegation and deconcentration. The study emphasized that the policy should include what is to be devolved and what is not. Also, Ghana was to develop the legal framework for health systems decentralization. The report stated that it was important for the policy to help enhance greater accountability, by adopting some of the mechanisms already in place, such as the District Assemblies Common Fund (DACF) to consolidate the various funds flows through integrated planning and budgeting processes, integrated M&E, developing equalization/equity formula and the performance-based financing mechanisms. Furthermore, local authorities could be given more control over budget/expenditure as well as the establishment of a clearer staff role and functions, and lines of authority within the District Assemblies and District Health Management Teams (10).

A Study into the Utilization of the District Assembliesʹ Common Fund (DACF) done in the New Juaben Municipality and published in 2013 revealed that the Assembly does not receive the total amount of Common Fund budgeted for in each year. In 2008, 46.67% of the Common Fund was received. In 2009, 51.33% was received while 40.75% of the Common Fund was received in 2010. They also observed that Education received the highest percentage of the Common Fund allocation of 22.45% whiles Health had 21.40% of the Common Fund allocation being the second highest sector in terms of the allocation of the common fund. Water and sanitation followed as the third sector with 16.75% of the common fund allocation. Agric sector and other departments of the District Assembly received smaller proportions of the DACF allocation (11).

Districts in Ghana are governed by District Assemblies, which are established by the Minister of Local Government, and serve as the highest political authority and decision-making body in each district. The membership of the District Assembly comprises: the District Chief Executive, appointed by the President of the Republic, one person (an Assemblyman or woman) from each electoral area within the district elected by universal adult suffrage, the member or members of Parliament from the constituencies that fall within the area of authority of the District Assembly, and other members that shall not exceed 30% of the total membership of the District Assembly appointed by the President in consultation with the traditional authorities and other interest groups in the district (12).

The District health systems of Ghana consist of networks of primary care health facilities that deliver a comprehensive range of promotive, preventive and curative health care services to a defined population with the active participation of the community and under the supervision of a district hospital and District Health Management Team. District health services are further organized into three levels: CHPS zone (community), subdistrict (health centres, clinics) and district (district hospital and district health directorate) (13).

Both the Local Government Act (462) of 1993 (14) and the Ghana Health Service (GHS) Act 525 of 1996 (15) have implied devolution (or political decentralization) of health at the district level to local government (district assembly). To date, the mode of decentralization for health remains administrative (deconcentration) rather than political (devolution). District Assemblies support for the District Health Systems have not been systematic. Relationships between the District Health Management Team (DHMT) and District Assembly (DA) remain *ad hoc* and personality-dependent (16). The financing pattern for health by local government appears fragmented and confusing (17).

**Figure 1:**
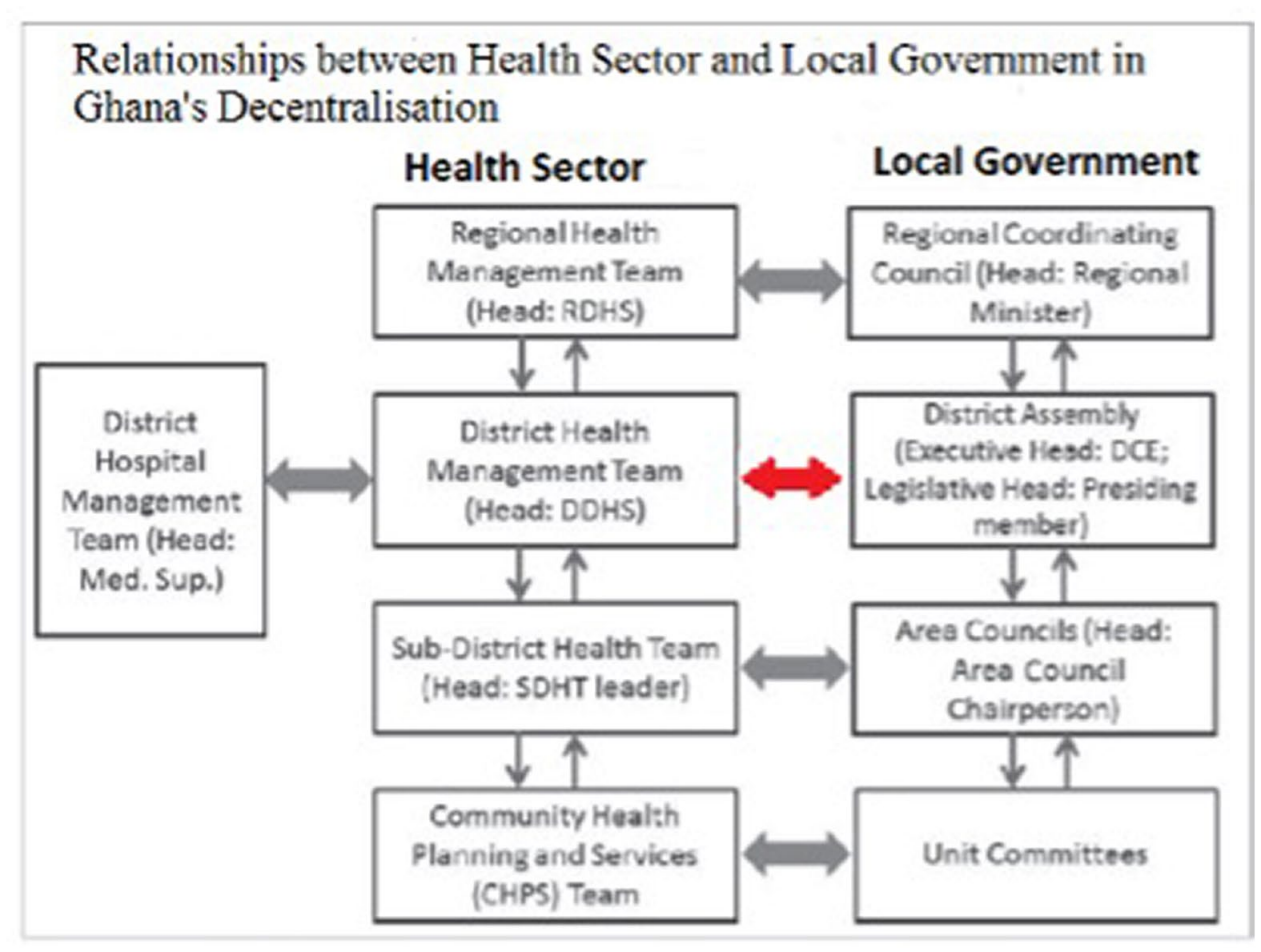
Relationship between local government and Ghana health sector in Ghana’s decentralization.

In the current administrative decentralization model, the district health system operates a matrix organizational structure reporting horizontally to the District Assembly and vertically to the Regional Health Directorate (Figure 1). There was delegation from the Ministry of Health to the Ghana Health Service (GHS) by Act 525 in 1996 and deconcentration from the headquarters of GHS to the Regional Health Directorates and District Health Directorates. The Ministry of local Government has deconcentrated to the Regional Coordinating Council and then to the District Assemblies. Greater power and influence appear to dominate the relationship between district and regional levels of the Ghana Health Service, compared to district health teams and local government (16, 18).

Ghana’s health sector is mainly financed by the central government, development partners, and private out of pocket payments by individuals and households. Public resources are allocated to the Ministry of Health (MoH) and health facilities through budgetary transfers, while the National Health Insurance Scheme (NHIS) is funded by the National Health Insurance (NHI) levy and by Social Security and National Insurance Trust (SSNIT) deductions (19). The Districts Assemblies financial obligations to the district health system in the context of decentralization is exercised through the District Assembly Common Fund (DACF), Member of Parliament’s (MP) Fund and Internally Generated Fund (IGF).

**Figure 2:**
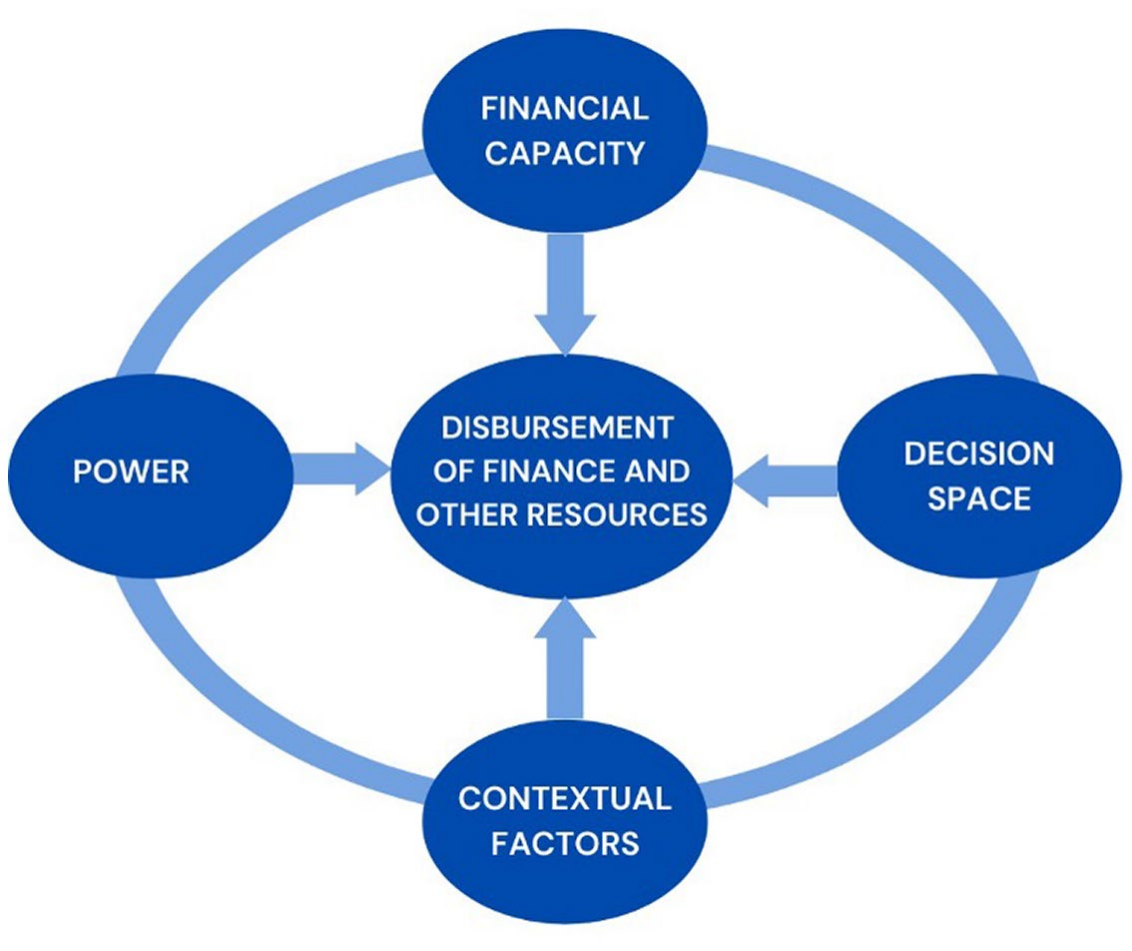
Contextual framework of factors influencing the disbursement of financial and other resources by the district assembly to the district health system.

The latest local government law Act 936 (Local Government Act 2016) in its intent suggests devolution in the health sector with the district health system becoming the health department of the District Assembly (section 77 (1) first schedule and section 78 (2) third schedule). Implementation of this law will imply that the department of Health of the District Assembly shall therefore perform the functions assigned to them under the local Government Instrument, 2009 (L.I 1961) and any other enactment in force (Act 936, section 80). Ghana is moving toward implementation of this Local Government Act, 2016 (Act 936) and other policies for the devolution of the district health system. In the past, decisions of District Assemblies to allocate and actual disbursement of financial and other resources to support the District Health System have been erratic and non-systematic (17). In this context it is important to understand the actual patterns of District Assembly financial and other resource support to the district health system, as well as the “why” of these patterns (Figure 2).

**Figure 3:**
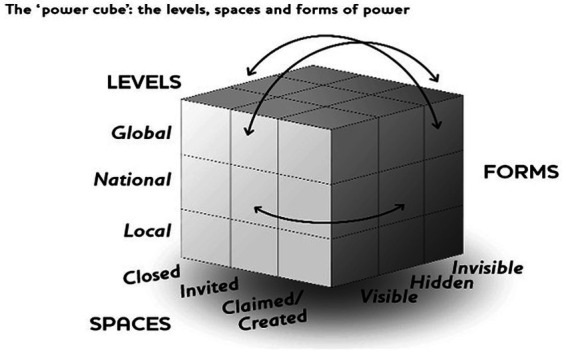
The ‘power cube’- The levels, spaces and forms of power (Reference: finding the spaces for change: a power analysis. IDS bulletin volume 37 No 6. November 2006. Institute of development studies).

### Research questions

The research questions were therefore: what are the sources of funding for district assemblies in Ghana; how much of these resources do these assemblies disburse to support the district health system to deliver services. How are disbursement decisions made and what are the lessons for developing and implementing decentralization policies and programs in Ghana to support primary health care and Universal Health Coverage (UHC) attainment?

### Conceptual framework

As a framework within which to organize to collect and analyze data to answer the research questions we theorized that the disbursement of financial and other resources by the District Assembly to the District Health System will be influenced by (1) Financial capacity of the district assembly to disburse these resources (including availability of funds), (2) the decision space of the District Assembly under the model of decentralization in implementation (3) the power and use of power by stakeholders and (4) contextual factors.

The above conceptual framework was drawn from aspects of various frameworks. To explore financial capacity, we drew upon Aragon’s framework (20). For decision space we drew on Bossert’s framework (21), for power we drew on Gaventa’s power cube (22) and for contextual factors we drew on Leichter’s classification (23).

Aragón (20) has divided organizational capacities into ‘hard’ capacities such as ‘infrastructure, technology, finances and ‘soft’ capacities, such as the ‘human and organizational capacities, or social capital of the organization, including management knowledge and skills, organizational systems and procedures, and procedures for planning and evaluation. The study drew on the organizational systems and procedures to understand the District Assemblies’ financial capacity to execute the budget process and the ‘soft’ capacities that influence the disbursement of funds to the District Health System.

In the Bossert theory, the decision space captures the degree to which local officials make use of decision-making powers and is classified into narrow, moderate and wide in order of freedom to decide. The study drew on the finance component of decision space by officers of the District Assembly in terms of sources of financial resources, allocation from central government as well as their internally generated funds.

As depicted in Figure 3 below, John Gaventa’s power framework conceptualizes ‘three dimensions’ of power as the spaces, levels and forms of power using a cube with each dimension representing one side of the cube (22).

The study drew on the concept of John Gaventa’s power framework in terms of how well officers of the district assemblies were able to influence disbursement through the various power structures at national and local levels.

To explore Contextual factors, we drew on the classification by Leichter into Situational factors, Structural factors, Cultural factors and environmental factors (Reference: Howard M. Leichter. A comparative approach to policy analysis. Health Care policy in four nations. Cambridge University Press 1979).

### Materials, subjects, and methods

The study design was a multiple (two district) mixed method case study. The comparative study of the two districts was done to enable exploration and understanding of the phenomenon in two different contrasting settings or context.

In the social sciences, the case study design is a common approach to conduct an in-depth investigation of a phenomenon that allows the exploration of the phenomenon of interest in its real-life context. In this case study, the phenomenon of interest was defined as “the why of the disbursement of financial and other (non-financial) resources by local government to the District Health system.” The district was considered as the single case or unit of analysis.

Both case study districts are in the Volta Region of Ghana (For the sake of anonymity, the districts are labeled as District A and District B). They were purposively selected to be able to study, compare the phenomenon of interest in a relatively developed and less developed district. The selection was guided by the Ghana District League Table (DLT) Report 2017. The DLT is an index of the developmental status of districts and ranks all the districts in Ghana based on indicators of performance in six key sectors – health, education, sanitation, water, security and governance. These indicators are aggregated into a single index, and districts are ranked from highest to lowest performing. The DLT is a joint Center for Democratic Development (CDD)-Ghana and UNICEF Ghana project, implemented in collaboration with the Ministry of Local Government and Rural Development and the Office of the Head of the Local Government Service. The report ranked District A as low level (less developed) district and District B as middle level (relatively developed).

District A was carved out of a bigger district in 2012 by Legislative Instrument LI.077 and the historic administrative records of that part of the district was assigned to the ‘new’ district (records before 2012 was retrieved and reviewed for this study). District A is located in the southern part of the Volta Region. The total population as of the 2010 census was 59,411 with a growth rate of 3.5%. The district has a total land area of about 700kmsq and 78.3% of the total households are engaged in agriculture. The major crops cultivated include maize, cassava, rice, pepper and tomatoes. The lower Volta Basin passes through the district and creates the opportunity for fishing. According to the 2019 District development profile document, there is one (1) District Hospital, four (4) Health Centres, one (1) private clinic, fourteen (14) CHPS compounds, one (1) private maternity home and one (1) private clinic. There are 71 basic schools, 50 Junior High Schools and two Senior High schools in the district.

District B is also located in the southern part of the Volta Region with a total land size of 779square kilometers and is a relatively resource-rich district with higher local government revenue generation capacity because it contains a major border crossing point between Ghana and Togo. Thus, goods and passengers traveling use this border crossing. According to the 2010 population and Housing Census, the total population was 160,756 with a growth rate of 2% with a sex ratio of 88.9 males per 100 females. According to the District’s 2019 Development profile compiled by the District Planning Committee Unit (DPCU), there are 33 health facilities comprising one (1) Government hospital, three (3) private hospitals, two (2) clinics, nine (9) health centres, two (2) private maternity home and sixteen (16) CHPS compounds. There are 82 public basic schools, 43 recognized private basic schools, 4 public Senior High Schools (SHS), 1 private SHS and one private technical/vocational institute. The population derives its livelihood from agriculture, Salt winning, Trade and commerce. The border market is a commercial distribution centre for agricultural produce from various regions of Ghana. Some of the goods are subsequently exported to Togo.

Data was collected from July 2019 to March 2020 using quantitative and qualitative methods of data collection involving a desk review of administrative documents, secondary data analysis of financial and routine management information system data of the district assembly and the district health system and Key informant in-depth interviews. Routine data compilation forms were designed and used to extract data on budget request, actual receipts, and financial allocations for the District Assembly and District Health system from 2004 to 2018.

Twelve key officials of each district assembly and the district health systems who had worked in the district for at least a year or more were purposively selected for key informant in-depth interviews. These officials were deemed by the District Assembly and District Health Systems to be associated with receipt and disbursement of financial resources by the District Finance officers and District Health Finance officers. District assembly officials interviewed included the District Coordinating Directors, District Finance Officers, Planning Officers, Budget Officers, Internal Auditors, members of the District Planning Committee and Assemblymen who were members of the social services committee. Within the district health system, officials interviewed included the Director of Health Services, District Health Finance Officer, District Public Health Nurse, District Health Service Administrator and Disease Control Officer. Respondents work experience in the District Assembly System in Ghana ranged from 2 years to 26 years (Table 1).

### Data analysis and calculations

The transcripts of the key informant in depth interviews were manually analyzed for themes, commonalities, and contrasts drawing on conceptual framework for the study. Additional to the themes from the conceptual framework, the interview transcripts were also analyzed for any new and emergent themes from the data that were not in the framework. The influence of power was analyzed in terms of levels, spaces and forms using data from the in-depth interview and also from the desk review. The decision space for decisions on disbursement of funds was analyzed on a three-point ordinal scale and classified as narrow decision space, moderate decision space and wide decision space. This classification was adapted from Bossert’s work (24) on analysis of decision space. In the Bossert’s decision space theory, the decision space captures the degree to which local officials make use of decision-making powers. The width of local decision space depends primarily on the degree to which local government decision-makers are permitted to make a variety of financial decisions, as opposed to decisions being handed down. Decision space may be widened or narrowed from below, such as local decision-makers who make decisions regardless of what official rules say or make choices which capitalize to a greater degree on available options (2, 25).

Routine data was entered into an excel spread sheet and analyzed for patterns and trends. We analyzed what percentage of the budgeted total funds the District Assembly received and used it as a proxy of the decision space they were permitted to spend within. The description of the sources of financial and other resources of the District Assembly and the proportionate disbursements to decentralized departments in this study was used as a proxy to the financial capacity of the Assemblies to meet their obligations and mandates to support the decentralized departments.

For quality assurance we followed the guidelines on best practices for ensuring rigor in qualitative research (26) and have also referred to the criteria included in the 32-item consolidated criteria for reporting qualitative research (COREQ) checklist to guide our reporting of our study.

Ethical approval was sought from the Ghana Health Service Ethical Review board (GHS-ERC No. 001/04/19) Permission was sought from the District Assembies and District Health Directorate of District A and B, the Volta Regional Coordinating Council and Volta Regional Health Directorate. All primary data was collected with informed consent. Each key informant was given a written study information sheet and a verbal explanation of the consenting process. A written consent form was signed by key informants before they took part in the study.

## Results

### What are the sources of funding of the district assemblies?

Three sources of financial resources of the two District Assemblies were identified namely: transfers from Central Government that can be from either general tax funding or centrally pooled donor funds; Internally generated funds (IGF) that are mobilized locally by the assemblies from citizens through taxation and other fees and Decentralized development partner funding sent directly to the assemblies with no central government transit.

The two funding pools from which transfers from Central Government are made to district assemblies are the District Assembly Common Fund (DACF) and the District Development Facility (DDF). The District Assemblies’ Common Fund (DACF) is a pool of resources from central government tax revenue created under Article 252 of the 1992 constitution of Ghana which requires a minimum of 5% of national revenue to be set aside to be shared among all District Assemblies in Ghana with a formula approved yearly by Parliament. The District Development Facility (DDF) is a donor pooled fund. In 2008, the Government of Ghana (GoG) and the governments of Germany, France, Canada and Denmark joined hands to establish DDF as a central donor pooled fund that is then disbursed by government to the district assemblies. IGF is revenue that is directly generated from citizens by District Assemblies (DAs) within their area of jurisdiction from local government taxes and other fees.

**Figure 4:**
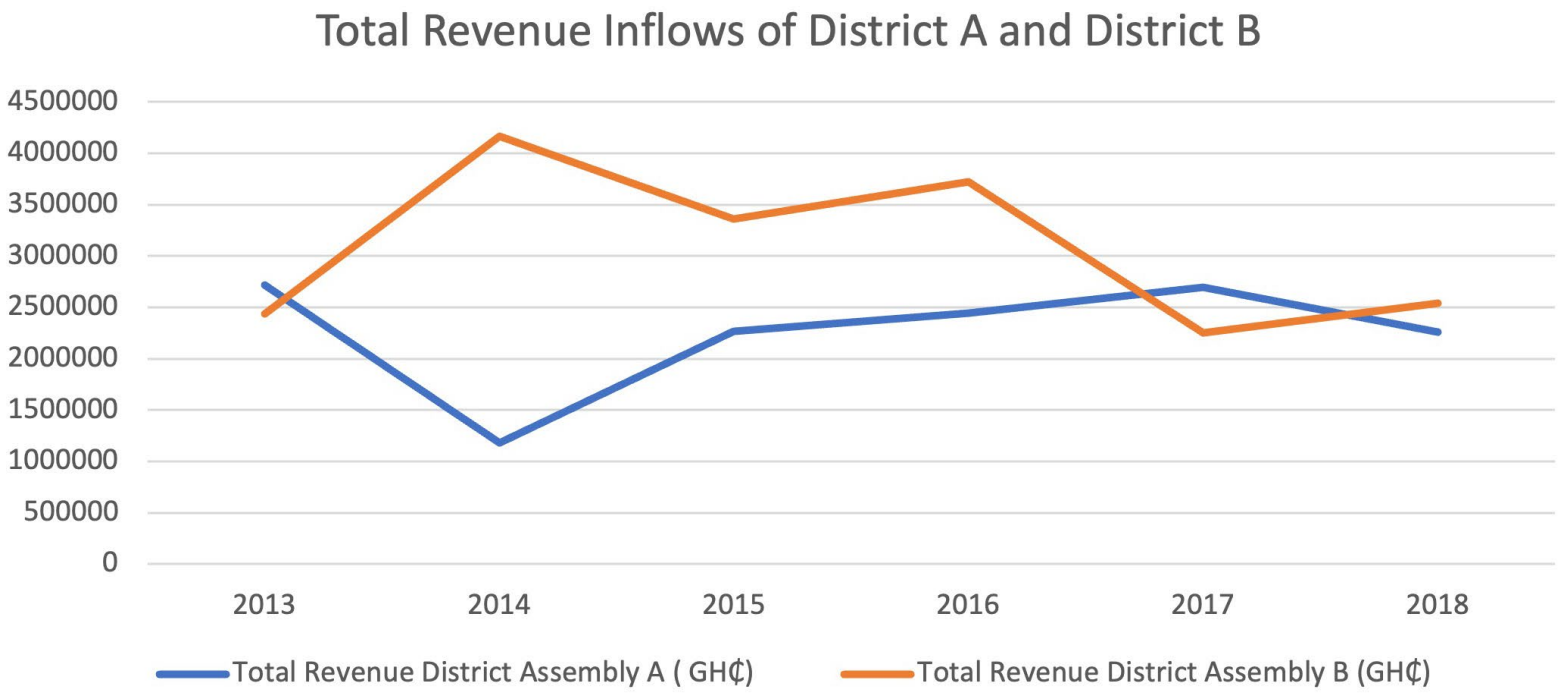
Total revenue inflows and the inflows by source for the two districts over the five-year period 2013–2018.

Figure 4 above shows the total financial resources that was received by the two study districts assemblies (A and B) from 2013 to 2018 and displays the irregular financial inflows. District B received more resources than District A from 2014 to 2016. In 2017, District A received more financial resources. District Assembly A experienced a decrease in financial resources from 2017 to 2018 whiles District B had a marked decrease in 2016 to 2017.

Also, analyzing the *per capita* District Assembly financial resource gave another difference between the financial capacity of the DAs. The population of District A in 2018 was 71,331 and the total financial resources was GH¢ 1,929,836.19. Therefore, District A’s *per capita* financial resources for disbursement was GH¢ 27.05 whiles District B’s *per capita* financial resources was GH¢12.82 because their 2018 population extrapolated from the 2010 census was 198,459 and their total revenue was GH¢ 2,544,864.35.

**Figure 5:**
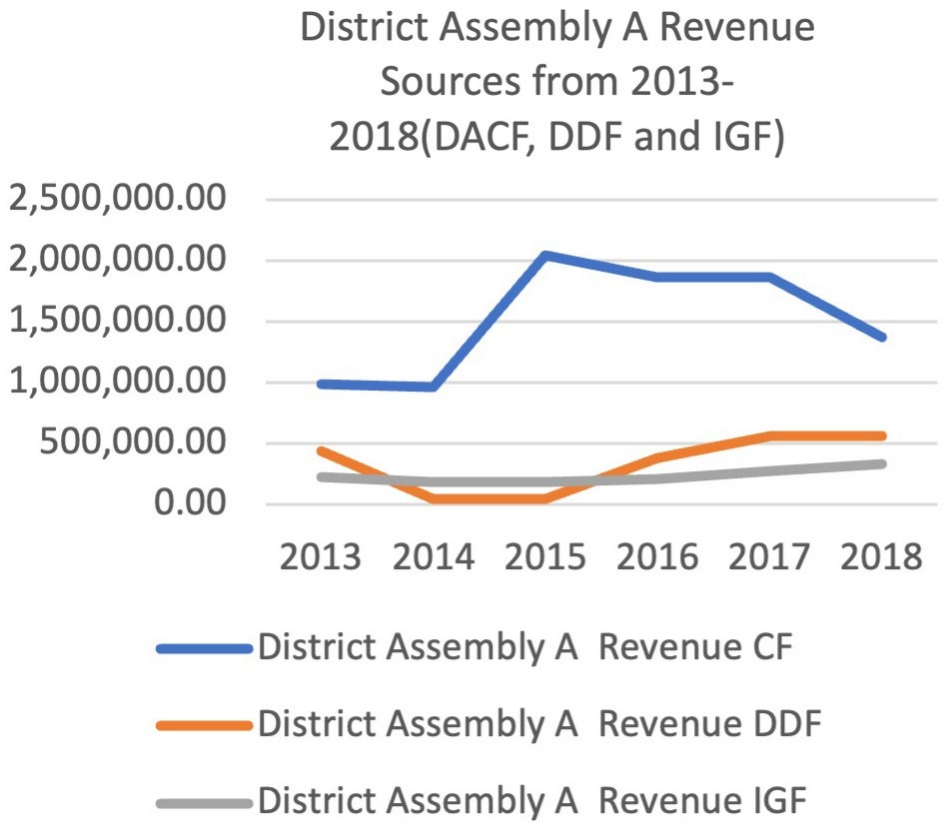
Ketu south municipal and central Tongu district revenue sources from 2013 to 2018.

**Figure 6:**
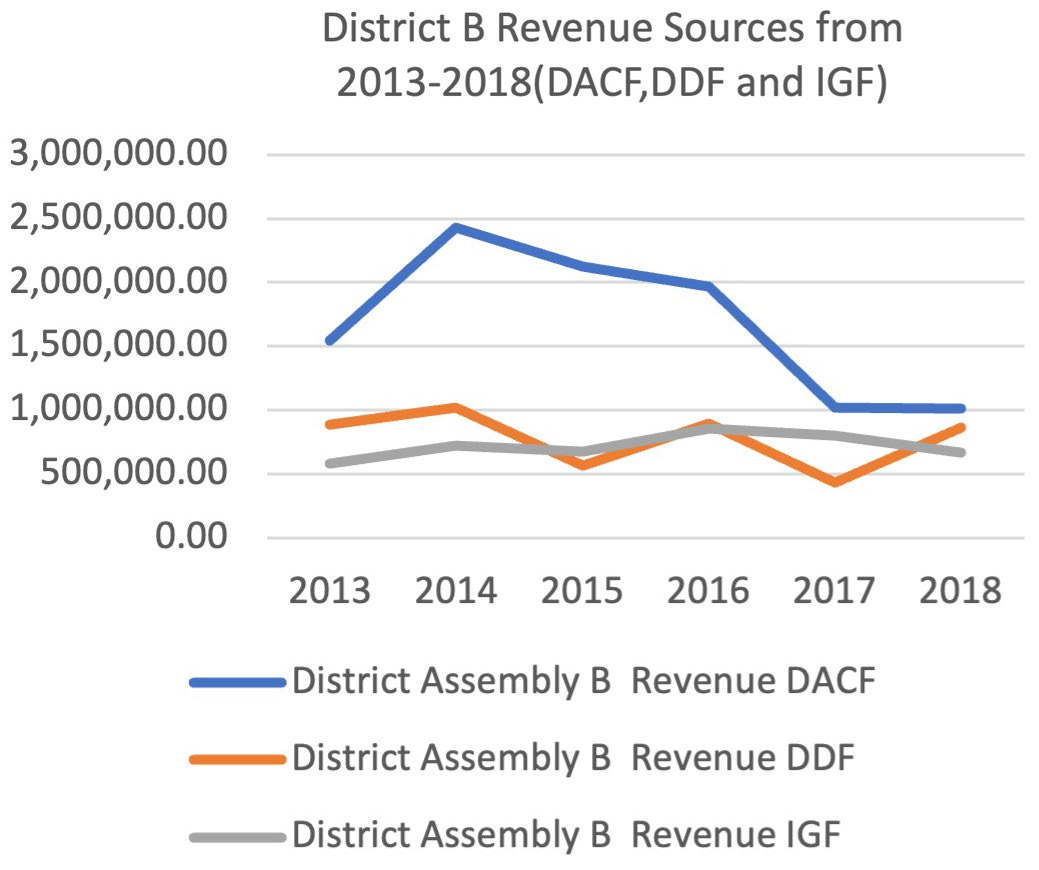
Percentage of budget received by district A and district B from 2013 to 2018.

Figures 5, 6 shows the relative levels of the different sources of financial resources to the district Assemblies. The two districts received financial resources from the three sources. The District Assembly Common Fund (DACF) was the highest financial resource to the two District Assemblies. District B received higher amounts for DDF and IGF from 2013 to 2018. In 2017 and 2018, District B’s DACF was generally higher than District A. The trend for sources from central government (DACF and DDF) was irregular from 2013 to 2018. The trend of IGF seemed relatively consistent from 2013 to 2018 in both Districts.

**Table 1:** Respondent demographic data.

Variable	District A	District B	Total
Number	12	12	24
Mean age (years)	49 years (Range32-65)	47 years (Range 31–55)	
Mean number of years of work in district assembly/district health system	4.2 years (Range 2–8)	4.2 years (Range 2-7 years)	
Mean number of years of work in the district assembly/district health system of ghana	10.4 years (Range 3–25)	13 years (Range 2–26)	
Male	11	11	22
Female	1	1	2
Groupings			
Officers of the district assembly	5	6	11
Officers of the district health system	4	5	9
Assemblymen	3	1	4

### How much of these resources do DA disburse to support the programmes of the DHS?

Tables 2, 3 shows how much of the District Assemblies’ financial resources was disbursed to the District Health System. The proportion of the total District Assembly financial resources was irregular for both District Assemblies. The proportion per year was also different in the two study districts. Funding of programmes of the District Health Systems (DHS) was limited and erratic in both districts (see Tables 4, 5). The better resourced District Assembly B provided more support to the district health system than the deprived District Assembly A. District Assembly A did not fund any programmes of the District Health System, except for HIV activities which was carried out by the Social Welfare department in collaboration with the District Health System. District Assembly B funded several of the programs of their DHS. The financial support ranged from 3 to 16.9% of the total cost of the programs. Majority of the programs funded by District Assembly B from 2004 to 2018 were programs initiated at the national level (e.g., National Immunization Campaigns, National Malaria Long-lasting bed net distribution campaigns).

**Table 2:** District A assembly total revenue and amount disbursed to district health system.

District A assembly revenue (GH¢)					Amount disbursed to district health system health	% disbursed to district health system health ghc
	DACF	DDF	IGF	Total		
2013	2,065,985	433,280	219,836	2,719,101	270,202	10%
2014	957,087.01	42,720	180,000	1,179,807.01	437,667.28	37%
2015	2,046,574.57	42,720	180,000	2,269,294.57	437,667.28	19%
2016	1,861,218.58	378,720	203,241.20	2,443,179.78	258,667	11%
2017	1,865,485.72	555,675	274,642	2,695,802.72	1,128,262	42%
2018	1,373,832.19	555,675	329,670	2,259,177.19	1,053,862	47%

**Table 3:** District B assembly total revenue and amount disbursed to district health system.

District B assembly revenue (GH¢)					Amount disbursed to district health system health	% disbursed to district health system health
	DACF	DDF	IGF	Total		
2013	1,548,201.71	889,000.90	580.922.15	2,437,202.61	52,164.20	2%
2014	2,428,565	1,020,392.15	719,756.67	4,168,713.82	32,059	1%
2015	2,125,155.92	562,402	672,215.10	3,359,773.02	80,635.60	2%
2016	1,965,522.99	897,200	858,102	3,720,825.23	511,583.27	14%
2017	1,020,400	432,546.36	800,635.60	2,253,581.96	580,191.84	26%
2018	1,010,954.26	865,836.97	668,073.12	2,544,864.35	87,119.05	3%

**Table 4:** District assembly A’s disbursement for HIV programme from 2013 to 2018.

Year	District Assembly A Funding Support for HIV Programme from 2013–2018
2013	I,440
2014	1,689.19
2015	706.59
2016	1,302.75
2017	1,313.67
2018	1,348.82

**Table 5:** District assembly B’s financial support for programmes of the district health system from 2004–2015.

Year	Health programmes funded by the DA	Amount of funding support (GH¢)	Total cost of the programmes carried out by the district health system for the particular year (GH¢)	Percentage of the total cost the municipal assembly’s support to DHS programs
2004	NID (National)	2,100	25,365	16.9%
	Cholera (Local)	2,185		
2005	NID (National)	4,600	56,685	11.6%
	Malaria (National)	2,000		
2009	NID (National)	10,058.50	115,694.73	10.3%
	Research (local)	1,816		
2010	H1NI flu (National)	3,000	90,780.44	3.3%
2011	NID (National)	15,088.32	112,578.32	19.1%
	Cholera (local)	6,371		
2014	NID (National)	2,080	68,933.73	3%
2015	NID (National)	15,440	112,761.55	13.7%

NID, national immunization days; DA, district assembly.

The issue of District Assemblies disbursing limited and irregular support to the programmes of the DHS was corroborated by the results of the in-depth interviews.

*“When we requested for money for NID, they ended up giving GHS 500 only. How do we use such a meagre amount to purchase enough fuel for such activity? So, we don’t get financial support from the assembly*.” (Health Officer, Central Tongu District)

The District Assemblies’ main support to the District Health System were in infrastructure as shown in Tables 6, 7 below.

**Table 6:** District assembly A’s disbursement for district health system health infrastructure from 2007 to 2018.

Year	Community/facility	Budgeted cost of project (GH¢)	Amount disbursed by the district assembly (GH¢)	Percentage of budgeted cost disbursed by district assembly (%)
2007–2009	CHPS A1	33,339.57	33,339.57	100%
2011	CHPS A2	76,915.96	72,255.71	93.9%
2011–2013	Health centre A3	43,416.45	45,571.40	104.9%
2012–2013	CHPS A4	56,754.37	53,067.53	93.5%
2012	CHPS A5	30,426.60	10,563.91	34.7%
2012–2013	CHPS A6	45,488.14	44,797.03	98.5%
2016–2018	District hospital A7	258,880.75	78,732.11	30.4%
2017–2018	CHPS A8	239,534.57	174,000.00	72.6%
2018–2019	CHPS A9	249,914.00	208,671.70	83.5%
2016–2018	CHPS A10	32,620.00	32,620.00	100%

**Table 7:** District assembly B’s budget and expenditure on health infrastructure from 2012 to 2019.

Year	Community/facility	Budgeted cost of project (GH¢)	Amount disbursed by the municipal assembly	Percentage of budgeted cost disbursed by district assembly (%)
2015–2017	CHPS B1	184,300.73	184,300.73	100%
2016–2018	CHPS B2	179,339.64	192,217.05	107.1%
2012	CHPS B3	85,385.33	11,972.19	14%
2012	CHPS B4	87,183.35	12,221.54	14%
2012	CHPS B5	86,130.52	12,078.67	14.3%
2012	CHPS B6	85,662.95	12,247.50	14.3%
2015–2017	CHPS B6	181,964.90	112,629.01	61.9%
2015–2019	CHPS B7	177,320.33	136,000.00	76.7%
2016–2018	CHPS B8	110,622.54	96,241.36	87%
2014–2019	CHPS B9	94,333.58	89,616.75	94%
2009–2012	CHPS B10	58,603.90	52,532.27	89.6%

Key informants explained that the community demand for health services were mostly construction of health facilities. It was not always clear to service providers how decisions as to where to site the clinics were finally arrived.

**Table 8:** Percentage of district assembly A’s revenue disbursed to health, education, and sanitation departments from 2013 to 2018.

Year	Health	Education	Sanitation
2013	10%	10%	11%
2014	37%	25%	28%
2015	19%	14%	6%
2016	11%	21%	11%
2017	42%	23%	10%
2018	47%	28%	12%

**Table 9:** Percentage of district B’s revenue disbursed to health, education, and sanitation departments.

Year	Health	Education	Sanitation
2013	2%	4%	31%
2014	1%	2%	14%
2015	2%	18%	17%
2016	14%	23%	10%
2017	26%	41%	20%
2018	3%	12%	4%

*“In terms of support, we get infrastructural support like CHPS compounds (which they put up at places which sometimes we don’t want). Apart from that, there is no financial support. I don’t think health should go under the Assembly*.” (Health Officer, District A)

Most infrastructure projects took 2–3 years to complete because of inadequate financial resources and some had to adjust the original cost to accommodate inflation. Others were left uncompleted because other priorities took up the resources of the District Assembly. The DACF and DDF were the main sources of funding for these projects. Participants explained that political gains were what motivated the District Assemblies to initiate more projects than their funding sources can support.

*“Sometimes, the Assembly is seen doing more projects for political gains but it is not the best. It is better to build 2 CHPS and pay upfront rather than spreading many projects which cannot be paid for, early”.* (District Assembly officer, District B)

District Assemblies (DA) did not specifically disburse financial resources for Maternal Newborn and Child Health (MNCH) related services which was one of the services the study expected the Assemblies to prioritize in terms of programmatic funding. Officers of the Assembly interviewed said they believed that every disbursement to health was a disbursement for MNCH related services since investments such as CHPS compounds, emergency ward and model schools for girls all ultimately benefitted MNCH.

*“We have a lot of maternal and child health problems in the communities. That is why we are now shifting our attention from education infrastructure to health. I am spending more on health than education. I want to at least construct not less than one CHPS compound in every single electoral area. That is my target now*.” (District Assembly Officer, District A)

Tables 8, 9 shows the irregular patterns of disbursements to the two study districts. In 2017 and 2018, District A disbursed more of its resources to Health whiles District B disbursed more of its resources to Education.

### How are disbursement decisions made?

The local government budget process is influenced by the needs of the communities as well as the prioritization process based on the central government’s budget ceiling given to the District Assembly. The District Planning Committee Unit (DPCU) which comprises officers of the District Assembly, Chairpersons of Committees of the District Assembly and Heads of Departments coordinate the process.

The budget process, which was similar in both study districts, began with the DPCU in the Assemblies engaging the communities in meetings with citizens to gather information on their needs.

*“We do community durbars, town hall meetings just to inform them and most of the time, you will hear the honorable DCE on radio. Almost every week community radio sensitizes the community on what the assembly is doing and our next plan”* (Assembly Officer, District A)

The heads of various departments were also asked to bring their departmental needs as inputs into the budget process. These inputs were fed into the development of four-year medium-term plans within the ceilings received for their budgets from central government. The needs were prioritized to fit into the ceiling given by central government. The budget developed was sent to the Finance and Administration subcommittee for scrutiny before been submitted to the Executive Committee of the District Assembly. From there, it was sent to the General Assembly for approval of the final budget, spelling out how the Assembly intended to disburse the financial resources.

*“Before we do our medium-term development plan, there is need assessment where we go to the communities and do stakeholder consultations. At times, their needs are more than necessary so we have to prioritize the community needs”.* (District Assembly Officer, District B)

The Central Government and local political leaders were very influential at the prioritizing stage of the budget process. The need for choices to be made generated the intense lobbying and the use of power to prioritize the budget items. The prioritizing process resulted in ‘elite capture” of projects in some instances despite efforts to make the process as objective as practicable. The percentage of the revenues of the District Assemblies disbursed to health in relation to two major departments of the DAs revealed an irregular and inconsistent disbursement pattern from 2013 to 2018 as shown in Tables 5, 6 below. In 2018 for instance, 47% of District Assembly A’s financial resources was disbursed to Health, whiles District Assembly B disbursed 3%. In that same year (2018), District A disbursed 28 and 12% to education and Sanitation departments, whiles District B disbursed 12 and 4%, respectively.

Key Informants mentioned that in spite of this theoretical structured process, the District Assembly’s disbursement was influenced by political factors such as the ability of stakeholders to lobby the District Assembly authorities.

*“You channel your lobbying through the Social Services Committee (SSC) to the Executive Committee (EXECO) and through the EXECO to the General Assembly. But if the General Assembly approves and you need the work to be done, then you have to lobby the DCE so that he can release the funds. The same applies to the MP’s fund”* (Health Officer, District A)

*“After you have finished preparing your budget, the government of the day will choose what the Common Fund should be used for. For instance, maybe the government needs 3 classrooms or CHPS compounds or a number of bore-holes. So, this can influence the budget we have prepared already because the project that the government is proposing might not be in the budget, so you have to drop some to suit the needs of the politician. So, when it happens like that, we roll it over to the following year*.” (District Assembly Officer, District B)

*“Yes, politics play a part in it. The politicians know their stronghold. Being a traditional leader, I have a fair idea of the communities’ needs and the distribution. So, when it comes to prioritization. We know which areas to prioritize. We don’t have the power to stop them (politicians) but we can lobby”.* (Assemblyman District A)

Occasionally, situational factors like rainstorms and security issues influence the capacity of the DAs to disburse resources. This was confirmed by participants in the study:

*“Maybe there is a rainstorm somewhere or a CHPS compound unroofed by rains. With that one, we have to get some money to them. We usually go for some contractors who can pre-finance it when such things happen and we pay afterwards”* (Assembly Officer, District A)

*“let’s say if there’s security issue right now, we have to deal with it till it is over, because if we allow it to escalate, then it means our work in the district will be affected”* (Assembly Officer, District B)

### Decision space, power, and disbursement of financial and other resources

The three sources of funding to the district assemblies as shown in Figures 5, 6 reveals that the District Assembly Common Fund (DACF) received from central government formed the highest source. Also, the District Development Facility (DDF) is a donor pooled fund allocated by central government. The internally generated fund which was within the control of the district assemblies was meagre. The officers of the District Assemblies (DAs) had no power over how much money they received from the national level and their receipts did not depend on how much they needed to deliver services to their departments or communities, but rather how much the national level had to offer. The district assemblies’ control over their revenue sources and central government allocations for their budgets was limited. Though decision space involves a complex determination of how many choices over different and the use of funding local officials are allowed (Bossert) the study decided that if receipts from central government was less than 25% of the DAs’ budget, their decision space will be determined as narrow. If receipts are more than 75% of their budgets, their decision space will be classified as wide. If it falls in between 25 and 75%, the decision space will be determined as moderate. From the study, District A received 44.7–75% of the budgeted funds from Central Government between 2013 and 2018 whiles District B received 42–83% in the same period (Figures 5, 6). This placed District A in the moderate decision space classification from 2013 to 2018 except for 2016 when 75% of their budget was realized, hence the district was classified in the wide decision space category for that year. District B experienced moderate decision space for 2013, 2014, 2017, 2018 and had 77 and 83% of their budget in 2015 and 2016 respectively, classifying their decision space as wide in the 2 years. The reduction of 30–41% in requested financial resources received by the District Assemblies in addition to a very irregular inflow. Adversely influenced their decision space. This assertion was expressed by participants.

*“Most times they serve us with a letter, indicating that this is our money, for this Assembly for this particular quarter. We don’t have any say*.” (Assembly Officer, District B)

*“Normally, the government brings what we call budget guidelines and, in the guidelines, they give us the ceiling*.” (Assembly Officer, District A)

*“Sometimes we have two or three quarters arears”* (Assembly Officer, District A)

The inadequate budgets and irregular flows rather than the felt needs of citizens determined how much of the local needs the District Assemblies were able to meet in a particular year.

## Discussion

Our findings suggest that even when summed up, the revenue from the three major sources of funding for district assemblies in Ghana is inadequate. Resource flows are moreover irregular and unpredictable. Though a formal process of developing district plans and budgets that involves consultations with communities and the decentralized departments occurs, the final budgets received l are only 42–83% of what is needed to execute these plans. This constrains decision space. The formal budget process are interrupted with the influence of powerful actors resulting in irregular and uncertain expectation of financial resources at the District Assemblies for disbursement. Moreover, apart from the formalized prioritization processes, in the context of multi-party democracy, political processes and lobbying also influence final disbursement decisions. Funding of the district health system by the local government is limited.

Central Government transfers which comprised the District Assembly Common Fund (DACF) and the District Development Facility (DDF) formed the major part of the financial resources of the district assembly. The amount of money from these two sources was consistently lower than the resource needed to fully finance district developmental and service delivery plans including health. Also, the total amount of District Assembly Common Fund allocated by Parliament influenced the capacity of the Assembly to disburse financial resources. This amount is prescribed by law, with Article 252 of the Constitution of Ghana stipulating that ‘not less than 5 % of the total revenues of Ghana’ should be shared among the districts.

Locally generated revenue (IGF) was limited in both districts and even more so in the less developed District A. The capacity of the District Assembly to compliment the Government transfers was limited by their inability to collect enough local generated revenue (IGF) from the citizens in the district. This made it difficult for the District Assemblies to adequately support District Health Systems.

The District Assemblies did not appear to have much power to influence the central government on the issues of budget ceilings for central funds. At the same time the limited local revenue generation (IGF) does not confer enough power for the District Assemblies to challenge the dictates of the central government even if they ‘do not agree’. These findings are similar to studies done in Kenya in which sub county managers experienced loss of autonomy and resources in a paper by Nyikuri et al. titled “We are toothless and hanging, but optimistic” (27). The study into the Utilization of the District Assembliesʹ Common Fund (DACF) done in the New Juaben Municipality and published in 2013 revealed that the District Assembly does not receive the total amount of DACF budgeted for each year. The findings in that study were that the DACF received in 2008, 2009, and 2010 were 46.67, 51.33, and 40.75% (11). The phenomenon of inadequate resource flow from central government is therefore a long standing issue that significantly influences the ability of the district assemblies to disburse enough resources to its departments and decentralized units.

The possibility of policy elites and officials capturing resources and the democratic process has long been a concern in democratic theory of decentralization. This is because the shift of power from the centre to the periphery is premised on the belief that local democratic institutions are the key to people being able to govern themselves but this is not wholly true (28). According to Ahwoi (29), one reason that accounts for this is the fact that the District Chief Executives are appointed by the president and therefore makes them defenders of central Government priorities rather than advocates of local priorities and interest (29). Also, Chiefs and traditional authorities, by virtue of their virtual exclusion from the local government system, have been rendered ineffective for forcefully articulating local priorities through the formal District Assembly processes of budgeting and prioritizing.

In line with the Bossert decision space approach, successful implementation of the devolution of the health system will depend on understanding the choice and decision space that is transferred from the central Ministry of Local Government to the District Assembly. It also depends on how much power is transferred to local officials of the District Assemblies to enable them exercise their discretion in disbursement of financial and other resources to the health department to run the needed health programmes (30). Hence the observation that the guidelines for the DDF and DACF created irregular patterns of moderate and high decision space for the officers of the District Assemblies was significant. The programmes that were national in nature like National Immunization Day campaigns (NID) in District B and HIV programmes in District A received support from the assemblies shows the influence of the national level on disbursement decisions to the District Health System. The DDF and DACF were mainly disbursed for infrastructure and capital expenditure. Contextual factors may serve as a source power to influence policy actors’ action, inaction, and choice (31). The resource constrained economic context of Ghana is undoubtedly part of the reason for the limited central transfers to the district assemblies as well as the challenges with additional local resource mobilization. The resulting reduction of 30–41% in financial resources in the budget that were actually received by the District Assemblies in addition to very irregular inflow limited decision space, hence decisions made based on the budgets were not carried out as expected.

If devolution of health to local government is to lead to improved performance of the district health systems and population health outcomes, decentralization policies should also focus on increasing central funds transfers to districts assemblies and support the District Assembly’s ability to generate substantial IGF. Additional to increased resource availability, there will be a need to strengthen the selection of what to fund with the resources. Also, there should be monitoring and evaluation of the impact of the programs and activities prioritized by district assemblies for funding on population health outcomes. It will be important to explore policies and strategies that will regulate the balance of power between community members and their representatives, politically appointed officers like the District Chief Executives (DCEs), technocrats and public servants and officers of District Assemblies in the decentralized departments and agencies. This can help to promote prioritization of disbursement decisions that respond more to local situational analysis and needs and limit undue political influence over the disbursement of central government allocations and Internally Generated Funds. Sub-optimal prioritization will further adversely affect the health and wellbeing of communities in the context of limited resources. Before the devolution of the District Health System to the District Assemblies using Act 936 (Local Government Act 2016) is implemented, the Ghanaian Parliament should open a dialogue with the District Assemblies about increasing the minimum of 5.0% of the national revenue set aside to be shared among all District Assemblies. Technical analysis should be commissioned to ascertain the minimum percentage that may be more reasonable in terms of moving forward human development, population health and well-being at district level to inform the debate. It will also be necessary to build capacity for district level officials and decision makers on local situational analysis, planning and selection of interventions for improving population health to help the assemblies look beyond infrastructure development and centrally initiated programs.

Limitations of the study.

We used the sources of finance and other resources of the District Assemblies as a proxy for the financial capacity and did not study the human resource skill mix and its influence on the decision-making processes on resource mobilization. This may have affected the interpretation of the relative amounts of the sources of financial resources of the District Assemblies that needed to be disbursed in particular ways. Also, we did not study the capacity of the District Health System to influence the disbursement of resources from the District Assemblies in terms of lobbying and relationship to the District Assemblies.

## Conclusion

The District Assemblies disbursed funds to the District Health Systems, but the high level of dependence on central government for financial resources associated with ceilings imposed on government allocations adversely influenced the power and decision space of the officers to disburse financial and non-financial resources to the District Health System. Also, though most of the budget was disbursed for infrastructure, there were reduced allocation of resources for services. Though the District Assemblies tried to be objective by involving its citizens in the selection of expenditure. Contextual factors like politics, economics, legal and situational factors working in an interrelating manner had significant influence as constraints as well as opportunities for disbursement of funds. This study confirmed the starting theorization that the disbursement of financial and other resources by the District Assembly to the District Health System was influenced by financial capacity of the District Assembly to disburse these resources, the decision space of the District Assembly under the model of decentralization, the power and use of power by stakeholders and context.

## Recommendations

The District Assemblies should be encouraged to support services and programmes of the District Health System aside the infrastructural support. Officers of the District Health System should be trained in lobbying skills to enhance their capacity to influence disbursement for services and programmes. Another important assessment that should be considered before the implementation of devolution of the District Health System is the readiness of the District Assemblies in terms of financial capacity, power relations and decision space available to officers as well as the effectiveness of the vehicle for articulating local needs through the formal budgeting systems.

## Data availability statement

The original contributions presented in the study are included in the article/supplementary material, further inquiries can be directed to the corresponding author.

## Author contributions

AA, IA, and NE: conception or design of work. AA: data collection and final approval of the version to be published. AA and IA: data analysis and interpretation, drafting the article, and critical revision of the article. All authors contributed to the article and approved the submitted version.

## Funding

The study was funded by the IDRC Canada Grant # 108237 – West and Central African Partnership for Maternal, Newborn, Child, and Adolescent health research also known as the Consortium for Mother Children Adolescent and Health Policy and System Strengthening (COMCAHPSS).

## Conflict of interest

The authors declare that the research was conducted in the absence of any commercial or financial relationships that could be construed as a potential conflict of interest.

## Publisher’s note

All claims expressed in this article are solely those of the authors and do not necessarily represent those of their affiliated organizations, or those of the publisher, the editors and the reviewers. Any product that may be evaluated in this article, or claim that may be made by its manufacturer, is not guaranteed or endorsed by the publisher.
